# Assessment of quality of life in patients with surgically treated maxillofacial fractures

**DOI:** 10.12688/f1000research.129579.1

**Published:** 2023-05-11

**Authors:** Sunil S Nayak, Srikant Gadicherla, Sreea Roy, Muskaan Chichra, Shriya Dhaundiyal, Vanishri S Nayak, Vinayak Kamath

**Affiliations:** 1 Department of Oral and Maxillofacial Surgery, Manipal College of Dental Sciences, Manipal, Manipal Academy of Higher Education,, Manipal, Karnataka, 576104, India; 2 Graduate Research Apprentice, Post grad, Northeastern university, Boston, USA; 3 Department of Anatomy, Kasturba Medical College, Manipal, Manipal Academy of Higher Education, Manipal, Karnataka, 576104, India; 4 Department of Public Health Dentistry,, Goa Dental College and Hospital,, Bambolim, Goa, India

**Keywords:** Quality of life, Maxillofacial injuries

## Abstract

**Background**: The complex nature of maxillofacial injuries can affect the surgical treatment outcomes and general well-being of the patient. To evaluate the efficiency of the surgical treatment, assessment of the quality of life (QOL) of the patients is of vital importance. Due to the absence of an exclusive QOL assessment tool for maxillofacial fractures, we introduce the ‘Twenty-point quality of life assessment in facial trauma patients in Indian population'. The aim of this study was to assess and evaluate the QOL following surgical management of maxillofacial trauma patients based on the severity of the injury.

**Methods**: The study consisted of 182 subjects divided into two groups of 91 each (Group A: severe facial injury and Group B: mild to moderate facial injury). The Facial Injury Severity Scale (FISS) was used to determine the severity of facial fractures and injuries. The twenty–point quality of life assessment tool includes Zone 1 (Psychosocial impact) and Zone 2 (Functional and aesthetic impact), with ten domains each to assess QOL.

**Results**: In Zone 1, the mean scores for Group A and Group B were 38.6 and 39.26, respectively. In Zone 2, Group B (44.56) had higher mean scores compared to Group A (32.92) (p< 0.001). Group B (83.8) had higher mean scores compared to Group A (71.58) when the total of both Zone 1 and Zone 2 were taken into consideration (p<0,001). In Group A, 9 out of 91 patients had a total score of 81- 100 compared to 68 in the same range in Group B.

**Conclusions**: Proper surgical management with adequate care to the hard and soft tissues can improve the QOL by reducing postoperative psychosocial and functional complications. Aesthetic outcomes play an important role in determining the QOL. Mild/ Moderate injuries show better QOL compared to severe maxillofacial injuries.

## Introduction

Maxillofacial trauma and associated injuries can cause severe physical deformity and psychological consequences. ^1^ Disability following major trauma has gained significant attention due to these related complications. ^2^ Poor quality of life in injured patients with a risk of developing psychiatric problems has been observed by some studies. ^3^^–^^6^ In the present era, to evaluate the impact of disease and the efficiency of the treatment, assessment of the quality of life of the patients is of vital importance. ^1^ Unfortunately, studies that assess the quality of life (QOL) after surgical treatment of maxillofacial fracture surgeries are rare and so we decided to investigate this.

Specific factors that influence the outcomes of trauma are the site of the fracture, the type of fracture, and the patient's age. ^7^ An associated fear factor with the treatment of fractures warrants the need to look into the psychological issues related to fracture surgery. ^8^^–^^10^ Though there are many quality of life (QOL) assessment tools in literature, an exclusive QOL assessment tool for maxillofacial fractures is lacking. We have devised an exclusive, first-of-its-kind QOL assessment tool that looks into both physical and psychological aspects following maxillofacial trauma surgery. The ‘Twenty-point Quality of life assessment in facial trauma patients in Indian population' assessment tool was developed and used to determine the QOL in this study. The aim of this study was to assess the QOL following surgical management of maxillofacial trauma patients based on the severity of the injury. The objectives of the study were:

1. To determine the domains that effect the QOL of a surgically treated individual.
2. To assess the efficiency of the surgical treatment based on the QOL results.

Also, the authors intend to introduce a new assessment tool to determine QOL precisely from a maxillofacial injury perspective.

## Methods

### Ethics and consent

The study was held in the Oral and Maxillofacial Surgery Department and Kasturba Hospital, Manipal, from January 2020 to October 2021 after approval from the Institutional Ethics Committee (IEC: 924/2019) on 19/11/2019. Written informed consent was obtained from each of the study participants.

### Participants

The study included patients between 18-70 years of age. The study participants were consecutive patients reporting to our unit with maxillofacial trauma who underwent maxillofacial fracture surgery. The patients were assessed 8-12 weeks after surgical intervention. A data schedule was prepared to document age, sex, and fracture type. The sex of the participants was determined based on self-report. A total of 188 patients were seen during the study period and were divided into 2 groups according to their consecutive arrival (Group A: severe facial injury and Group B: mild to moderate facial injury) based on the severity of maxillofacial fractures and facial injury. Six patients (three from each group) were lost at follow-up. The final study consisted of 182 subjects divided into two groups of 91 each. Patients with associated diseases like cysts or tumors of the jaw bones, pregnant women, and those with underlying psychological issues were excluded from the study.

### Instruments and treatment

We followed the Facial Injury Severity Scale (FISS) ^11^ to determine the severity of facial fractures and injuries prior to evaluating the QOL. The face is divided horizontally into the mandibular, mid-facial, and upper facial thirds. Fractures in these thirds are given points based on their type ( Table 1). Injuries with a total score above 4.4 were considered severe facial injuries (Group A), and those with a total score below 4.4 were considered mild/moderate facial injuries (Group B). The QOL was compared between the two groups.

**Table 1.** Facial injury severity scale (FISS).

Type of fracture	Points
**Mandible**	
• Dentoalveolar/Condyle/Coronoid	1
• Body/Ramus/Symphysis	2
**Mid-face**	
• Dento Alveolar	1
• Le Fort I	2
• Le Fort II	4
• Le Fort III	6
• Naso-Orbital Ethmoid (NOE) 3 points	3
• Zygomatico Maxillary Complex (ZMC) 1 point	1
• Nasal	1
**Upper face**	
• Orbital roof/rim	1
• Displaced frontal sinus/bone fractures	5
• Non-displaced fractures	1
**Facial laceration**	
• Over 10 cm long	1

Note: Unilateral Le Fort fractures are assigned half the assigned points. The FISS is the sum total of the above points in an individual.

Meticulous management of hard and soft tissue injuries in our state-of-the-art tertiary care hospital was implemented. All elective cases were surgically treated at least 72 hours after the initial trauma. The facial fractures were adequately reduced and fixed with high–end titanium miniplates and screws (AO Principles of Fracture Management). Soft tissue injuries were managed by wound debridement, removal of foreign bodies, and layered wound closure. Adequate pain-relieving medication was prescribed to the patients postoperatively for effective pain control.

The QOL of the subjects was assessed using the 'Twenty-point Quality of life assessment in facial trauma patients in Indian population' assessment tool. The development was based on the WHOQOL-BREF scale and was modified from a maxillofacial trauma point of view. The twenty-point QOL assessment contains 20 questions (domains) and uses a five-point Likert response scale, and includes two zones: Zone 1 (Psychosocial impact) ( Table 2) and Zone 2 (Functional and aesthetic impact) ( Table 3), with ten questions (domains) each. The scores for each question ranged from 1-5, with a higher score denoting better quality of life. Accordingly, the score in each zone for a patient ranged from 10-50, and the total scores of both zones were recorded to determine the QOL. The sum of both zones determined the prognosis following surgery ( Table 4).

**Table 2.** Zone 1- Psychosocial Impact in the 20-point quality of life assessment.

	Strongly disagree	Disagree	Neutral	Agree	Strongly agree
	1	2	3	4	5
**Questions**					
1. **You are satisfied with your overall physical health**					
2. **You can perform your daily activities without any medicines or medical/surgical aid**					
3. **You can concentrate on your life and enjoy it as same as before**					
4. **You are confident to mix with your friends and family in the same way as before**					
5. **You are able to get good sleep**					
6. **Your personal relations have not experienced a set back**					
7. **You are satisfied with the support you received from friends/family/colleagues during treatment and recovery phase**					
8. **You don’t have aversion or hatred towards life? You never had suicidal feelings**					
9. **You can accept changes in your facial appearance**					
10. **You are happy with the medical services provided to you during your treatment**					

**Table 3.** Zone 2- Functional and aesthetic impact in the 20-point quality of life assessment.

	Strongly disagree	Disagree	Neutral	Agree	Strongly agree
	1	2	3	4	5
**Questions**					
1. **You are happy with decline in pain level**					
2. **You are happy with the reduction in amount of swelling**					
3. **You can enjoy your daily meals like before**					
4. **You can open your mouth as wide and easily as before**					
5. **The surgical hardware does not cause you any irritation**					
6. **You can feel the same sensation of touch on your cheeks/chin as before**					
7. **You don’t feel any discomfort in swallowing**					
8. **You are completely satisfied the way you talk**					
9. **You feel that you look good as before**					
10. **Your vision is as good as before**					

**Table 4.** Analysis of the 20-point Quality of Life Assessment scores.

Total score of Zone 1 + Total score of Zone 2	Prognosis
81–100	Excellent outcome
61–80	Good outcome
41–60	Fair outcome
21–40	Bad outcome
20	Worst outcome

The Test-retest method was used to determine the reliability and the correlation coefficient ( *r*) values was above 0.7 The scale was piloted previously but 2 questions (1 each from each zone) were modified due to the poor understanding by the participants:

• Question 3: You can concentrate on you **r** life and enjoy it as same as before (the earlier version was ‘Can you focus well in your life?’)
• Question 13: You can enjoy your daily meals like before (the earlier version was ‘Are your tongue movements normal at present?’)

The data collected was entered into a Microsoft Excel 365 MSO (Version 2301 Build 16.0.16026.20002) 64-bit spreadsheet and analyzed in the form of frequency and percentage for categorical variables, and in the form of mean, median, standard deviation, and quartiles for continuous variables. A non-parametric test was used and QOL scores were compared using SPSS Statistics, Version 22 (Armonk, NY: IBM Corp). Descriptive data were presented in between the study groups using the Mann-Whitney U test. P value < 0.05 was considered statistically significant.

## Results

The study group comprises 182 subjects (145 males and 37 females) ( Table 5). 160 were below 65 years of age ( Table 5). The causes of fractures were road traffic accidents (84.3%), violence (5.3%), falls (8.2%), and sports activities (2.2%). In Zone 1, the mean scores for Group A and Group B were 38.6 and 39.26, respectively. In Zone 2, Group B (44.56) had higher mean scores compared to Group A (32.92), and this was statistically significant (p< 0.001) ( Table 6). Group B (83.8) had higher mean scores compared to Group A (71.58) when the total of both Zone 1 and Zone 2 were taken into consideration, and this was statistically significant (p<0.001) ( Table 6).

**Table 5.** Demographic details.

		Frequency	Percent
**Age**	65-70 years	22	12.1
	18-64 years	160	87.9
**Sex**	Male	145	79.7
	Female	37	20.3

**Table 6.** Comparison of quality of life between the study groups according to Zone 1 and Zone 2.

	Facial injury severity scale (FISS)	N	Mean	SD	Min	Max	Percentiles			Mann Whitney U Test	
							Q1	Median	Q3	U Statistic	p-value
**Zone 1**	**A**	91	38.6	7.13	25	50	31	40	45	3973	0.64(NS)
	**B**	91	39.26	5.00	29	50	36	38	43		
**Zone 2**	**A**	91	32.92	1.90	28	36	32	33	34	0	<0.001 *
	**B**	91	44.56	1.98	38	47	44	45	46		
**Zone 1 + Zone 2**	**A**	91	71.58	7.12	57	83	65	73	78	651	<0.001 *
	**B**	91	83.8	5.19	75	96	80	83	88		

* p<0.05 statistically significant. p>0.05 non-significant (NS), A=Group A, B=Group B.

**Figure 1.**
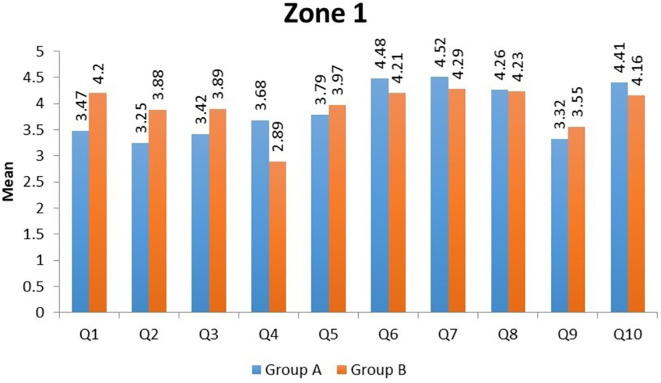
Mean scores of individual domains in Group A and Group B in Zone 1.

**Figure 2.**
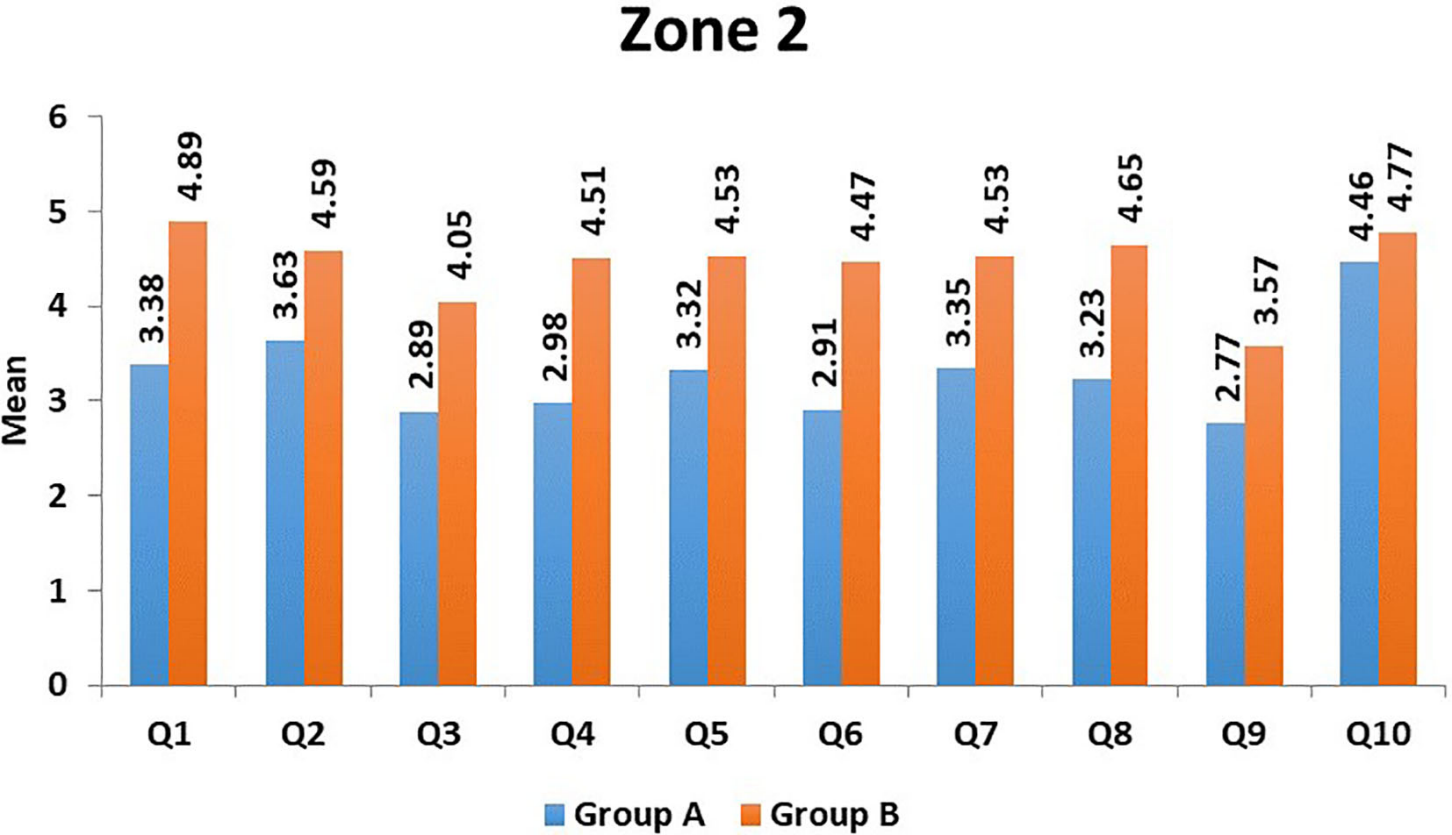
Mean scores of individual domains in Group A and Group B in Zone 2.

In Zone 1, the domains of 'Satisfaction in daily activities' (Question 2) and 'Acceptance of post-trauma facial appearance' (Question 9) had the lowest mean scores in Group A. In Group B, 'the ability to interact with family and friends' (Question 4) domain showed the lowest mean score ( Table 7, Figure 1). In Zone 2, the domains of 'aesthetics' (Question 9) and 'mastication' (Question 3) had the lowest mean scores in Group A, and in Group B, the 'aesthetics' domain (Question 9) had the lowest mean score ( Table 8, Figure 2). On comparison of individual domains between the two groups in Zone 1, except for domains of 'Quality of sleep,' 'Lack of suicidal tendencies,' and 'Acceptance of post-trauma facial appearance,' all other domains showed statistically significant differences ( Table 7). In Zone 2, on comparison of the two groups, all domains showed statistically significant differences (p<0.001) ( Table 8).

**Table 7.** Comparison of individual domains of Zone 1 between the study groups.

Zone 1	Facial injury severity scale (FISS)	N	Mean	SD	Min	Max	Percentiles			Mann Whitey U Test	
							Q1	Median	Q3	U Statistic	p-value
Q1 (Overall physical health satisfaction)	**A**	91	3.47	1.07	1	5	3	4	4	2547.5	<0.001 *
	**B**	91	4.20	0.79	2	5	4	4	5		
Q2 (Satisfaction in daily activities)	**A**	91	3.25	1.08	1	5	2	3	4	2778.5	<0.001 *
	**B**	91	3.88	0.95	2	5	3	4	5		
Q3 (Ability to concentrate)	**A**	91	3.42	1.08	2	5	3	3	4	3090	0.002 *
	**B**	91	3.89	0.92	2	5	3	4	5		
Q4 (Ability to interact)	**A**	91	3.68	1.27	1	5	3	4	5	2819	<0.001 *
	**B**	91	2.89	1.46	1	5	2	2	4		
Q5 (Quality of sleep)	**A**	91	3.79	1.15	1	5	3	4	5	3847.5	0.39(NS)
	**B**	91	3.97	0.97	2	5	3	4	5		
Q6 (Personal relationship status)	**A**	91	4.48	0.77	2	5	4	5	5	3393.5	0.02 *
	**B**	91	4.21	0.89	1	5	4	4	5		
Q7 (Family support during treatment)	**A**	91	4.52	0.71	3	5	4	5	5	3371	0.02 *
	**B**	91	4.29	0.72	3	5	4	4	5		
Q8 (Lack of suicidal tendencies)	**A**	91	4.26	0.89	2	5	4	5	5	3971	0.61(NS)
	**B**	91	4.23	0.83	1	5	4	4	5		
Q9 (Acceptance of post trauma facial appearance)	**A**	91	3.32	1.31	1	5	2	3	5	3732	0.24(NS)
	**B**	91	3.55	1.11	1	5	3	4	4		
Q10 (Satisfaction with the medical services)	**A**	91	4.41	0.65	3	5	4	4	5	3488	0.04 *
	**B**	91	4.16	0.79	2	5	4	4	5		

* p<0.05 statistically significant. p>0.05 non significant (NS), A=Group A, B=Group B.

**Table 8.** Comparison of individual domains of Zone 2 between the study groups.

Zone 2	Facial injury severity scale (FISS)	N	Mean	SD	Min	Max	Percentiles			Mann Whitey U Test	
							Q1	Median	Q3	U Statistic	p-value
Q1 (Pain)	**A**	91	3.38	0.68	2	4	3	3	4	225	<0.001 *
	**B**	91	4.89	0.31	4	5	5	5	5		
Q2 (Edema)	**A**	91	3.63	0.80	1	5	3	4	4	1454	<0.001 *
	**B**	91	4.59	0.49	4	5	4	5	5		
Q3 (Mastication/Chewing)	**A**	91	2.89	0.71	2	4	2	3	3	1021.5	<0.001 *
	**B**	91	4.05	0.55	3	5	4	4	4		
Q4 (Trismus)	**A**	91	2.98	0.76	1	4	3	3	3	612	<0.001 *
	**B**	91	4.51	0.62	3	5	4	5	5		
Q5 (Hardware failure)	**A**	91	3.32	0.54	2	4	3	3	4	688	<0.001 *
	**B**	91	4.53	0.50	4	5	4	5	5		
Q6 (Paresthesia)	**A**	91	2.91	0.66	2	4	2	3	3	511.5	<0.001 *
	**B**	91	4.47	0.60	3	5	4	5	5		
Q7 (Swallowing)	**A**	91	3.35	0.64	2	4	3	3	4	967.5	<0.001 *
	**B**	91	4.53	0.60	3	5	4	5	5		
Q8 (Speech)	**A**	91	3.23	0.65	2	4	3	3	4	512	<0.001 *
	**B**	91	4.65	0.48	4	5	4	5	5		
Q9 (Aesthetics)	**A**	91	2.77	0.63	1	4	3	3	3	1707.5	<0.001 *
	**B**	91	3.57	0.78	2	5	3	4	4		
Q10 (Ocular/visual disturbances)	**A**	91	4.46	0.60	3	5	4	5	5	3041.5	<0.001 *
	**B**	91	4.77	0.42	4	5	5	5	5		

* p<0.05 statistically significant. p>0.05 non significant (NS), A=Group A, B=Group B.

In Group A, 9 out of 91 patients had a total score of 81-100. Three patients scored below 60, and the remaining 79 scored in the 61-80 range. Two out of 16 female participants had excellent prognosis and the remaining 14 showed good prognosis. In Group B, 68 patients scored in the 81-100 range, and the remaining 23 scored in the 61-80 range. 14 out of 21 female participants showed excellent prognosis and the remaining seven showed good prognosis ( Table 9).

**Table 9.** Quality of life outcomes among groups.

Group A (n= 91)	Group B ( n=91)	Score	Prognosis
9 (7 M, 2 F)	68 (54 M, 14 F)	81–100	Excellent outcome
79 (65 M, 14 F)	23 (16 M, 7 F)	61–80	Good outcome
3 (3 M, 0 F)	-	41–60	Fair outcome
-	-	21–40	Bad outcome
-	-	20	Worst outcome

n=total number of study participants; M=males; F=females.

## Discussion

The inclusion of assessment of QOL is essential in treating patients with maxillofacial fractures to determine psychological well-being and patient satisfaction. ^12^ Unfortunately, the evaluation of QOL of people who had been surgically treated for maxillofacial fractures is not practiced routinely. ^13^ While assessing QOL in maxillofacial injuries, it is essential to consider the severity of the injury. Maxillofacial injuries occur in various combinations, and individual fractures require specific descriptions. We used the FISS to classify severe and moderate/mild injuries. The FISS is a valuable tool for maxillofacial trauma assessment. This scale can reliably predict the severity of maxillofacial injuries and is easily calculated. ^11^ We consider 4.4 as the average demarcating score to classify severe and moderate/mild injuries based on the study by Bhageri *et al.* ^11^

Several assessment tools are available to determine the well-being and QOL of patients, but maxillofacial injuries are unique due to the disfigurement and dysfunction they cause. These injuries can lower the person's self-esteem and adversely affect daily activities and social relationships, ultimately affecting QOL. ^13^^,^^14^ We have devised an exclusive QOL assessment tool for facial trauma patients to assess post-surgery patients from a maxillofacial trauma perspective. The new assessment tool determines both the psychosocial and functional and aesthetic impacts due to maxillofacial fracture surgery.

WHO's Quality of Life (WHOQOL-100) and its shorter version, the WHOQOL-BREF, are used in various settings to determine patients' quality of life. ^15^ The WHOQOL-BREF, more popular among the two, contains four domains: physical health, psychological health, social relationships, and environment. ^13^ Mood disorders, body image disorders, and a poor QOL are often exhibited in aesthetic and functional disturbances associated with maxillofacial trauma. ^16^ A disfiguring maxillofacial injury can make an individual withdraw from social interaction. ^1^ Also, a lack of support from family and friends affects an individual's physical and emotional well-being. ^16^ All these observations were considered in the new assessment tool (The Twenty-point Quality of life assessment in facial trauma patients in Indian population), which we devised. The psychosocial impact questionnaire evaluated the patient's satisfaction with their overall physical health, the confidence of the individual in performing daily activities and interacting with family and friends, and the ability to concentrate and sleep well (Zone 1, Questions 1-5) ( Table 2). Also, the personal relationships of the patient with people around them, the support by their near and dear ones, suicidal tendencies, if any, ability to accept changes in facial appearance, and satisfaction with medical services provided can be assessed (Zone 1, Questions 6-10). Hence the four domains of the WHOQOL-BREF evaluation tool are given adequate importance in the new assessment tool presented by the authors.

Moreover, we also determined the functional and aesthetic impact on subjects after maxillofacial surgery (Zone 2) ( Table 3). The progress of pain, swelling, mouth opening, and paresthesia can be assessed by this questionnaire (Zone 2, Questions 1, 2, 4, and 6). Evaluation of daily activities like eating, swallowing, talking (Zone 2, Questions 3, 7, 8), and discomfort due to the hardware (Zone 2, Question 5) are also evaluated. This assessment tool also considers patient concerns regarding facial appearance and visual disturbances (Zone 2, Questions 9, 10).

Previous studies have documented that patients with facial trauma subsequently exhibit poor QOL outcomes. ^17^^–^^20^ The inflammatory response caused by a facial injury results in increased vascular permeability, vasodilation, and infiltration of monocytes and polymorphonuclear leukocytes in the area of injury. These changes take place within a few days following an injury. Hence an early fracture surgery can lead to an unfavorable outcome due to a lack of initial blood supply. ^21^ In our study, most surgical treatment of fractures was done at least 72 hours after injury to achieve favorable outcomes.

Complications in all surgically treated patients accounted for 6.6%. Alcohol abuse, smoking, and plating procedures are some factors significantly associated with complications. ^22^ The findings of our study suggest that adequate reduction and fixation of fractures with high-end titanium miniplates and screw systems (AO Principles of Fracture Management) and efficient management of soft tissue injuries greatly enhance the outcomes of facial trauma patients. Our unit's soft tissue injury management included adequate debridement of devitalized tissue, layered closure of the wounds, and aesthetic reconstruction of soft tissue injuries with tissue loss. By following these measures, the outcomes of the surgical intervention can be significantly enhanced, as shown in our study. Moreover, severe injuries are associated with extensive soft tissue injuries, which can lead to poor QOL. In our study, the mean total scores (Zone 1+ Zone 2) show a good outcome for Group A subjects with severe injuries (71.58) and an excellent outcome for Group B subjects with mild/moderate injuries (83.8) ( Table 6). These favorable outcomes in our study can be attributed to the use of state-of-the-art titanium hardware for fixation, excellent soft tissue care, restoring the functional ability of the patient, such as chewing and mouth opening, and adequate control of postoperative pain and edema.

**Figure 3.**
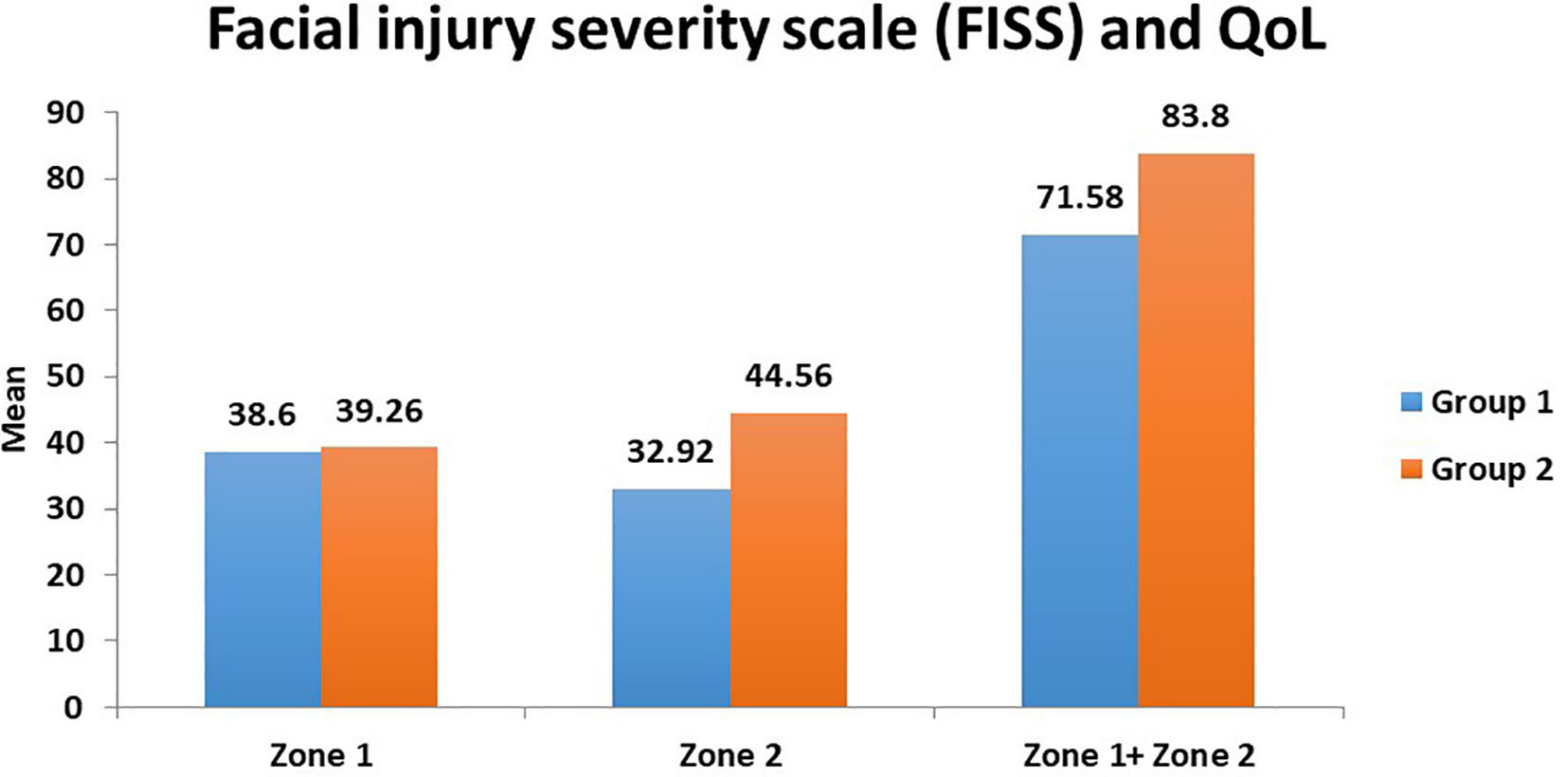
Relationship between severity of injury (Group 1 and 2) and quality of life (zones 1 and 2).

A lack of improvement in QOL after surgery was attributed to appearance, pain, and mood issues in the postoperative period. ^23^ Also, the primary concern of patients with maxillofacial trauma compared to other types of trauma is their appearance. ^24^ These findings correlate to our study in which the ‘facial appearance’ domain in Zone 1 and the ‘aesthetics’ domain in Zone 2 had low mean scores ( Tables 7 and 8). As both these domains are related to the cosmetic appearance of the patient, cosmetic defects can adversely affect QOL in patients. Severe maxillofacial injuries can cause cosmetic defects. Cosmetic defects caused by maxillofacial trauma can, in turn, lead to depression and affect QOL. ^25^ Moreover, trauma leading to difficulty in chewing and functional impairment such as paresthesia and diplopia can also cause depression in individuals. ^26^ Hence, a correlation exists between psychological and aesthetic/functional components resulting from severe maxillofacial trauma. These findings relate to our study wherein the QOL was comparatively better in mild to moderate injuries compared to severe ones. In our study, better QOL was seen in Group B (mild/moderate facial injuries) compared to Group A (severe facial injuries) ( Figure 3). There was statistical significance in Zone 2 scores and the combined Zone 1 + Zone 2 scores ( Table 6).

Patients who underwent open reduction and internal fixation of fractures recorded higher pain scores post-surgery. ^27^^–^^29^ Pain reduces the QOL of patients, and hence it is essential to employ adequate pain reduction protocols post-surgery to improve QOL. ^30^ Our study also shows the effect of pain on the QOL of the patient. The average mean score for decline in pain (Zone 2, Question 1) was 3.38 and 4.89, respectively, for severe and mild/moderate injuries ( Figure 2). As higher scores denote better QOL, it is evident that mild/moderate injuries show better QOL compared to severe injuries. Adequate analgesics are to be prescribed to patients for effective pain control postoperatively. Moreover, we believe that meticulous and gentle handling of soft and hard tissues during the surgical procedure is vital in decreasing postoperative pain, edema, and patient discomfort. Anggayanti *et al*. reported significant improvement in QOL within 14 days of surgical intervention. ^31^ Open reduction and fixation in association with excellent management of postoperative complications restore the normal configuration of anatomic structures, enhance stability, and establishes normal function. ^32^ Efficient surgical management, good postoperative care, and due consideration of facial aesthetics were employed in our unit to obtain favorable outcomes. Both sexes showed excellent outcome in Group B compared to Group A ( Table 9). The higher male:female ratio of the participants can be attributed to the fact that motorcycles in India are predominantly driven by males. Due to this higher male:female ratio, only a descriptive analysis based on sex of the individual has been demonstrated.

The study had certain limitations. As we had used a new diagnostic tool, its credibility could have been enhanced by advocating it to assess the pre-operative QOL and comparing the outcomes with the present findings. Also, comparing postoperative outcomes at different periods could have shed light on the possibility of improvement in the QOL over a period of time after surgery.

## Conclusion

Proper surgical management with adequate care to the hard and soft tissues can improve the QOL by reducing postoperative psychosocial and functional complications. Aesthetic outcomes play an important role in determining the QOL. Mild/Moderate injuries show better QOL compared to severe maxillofacial injuries.

## Data Availability

figshare: Quality of Life in Patients with Surgically Treated Maxillofacial Fractures, https://doi.org/10.6084/m9.figshare.21702023.v4. ^33^ This project contains the raw data file: QoL Life Data.xlsx figshare: Quality of Life in Patients with Surgically Treated Maxillofacial Fractures, https://doi.org/10.6084/m9.figshare.21702023.v4. ^33^ This project contains the ‘Twenty-point quality of life assessment in facial trauma patients in Indian population' questionnaire. Data are available under the terms of the Creative Commons Zero “No rights reserved” data waiver (CC0 1.0 Public domain dedication).
