# Supplementary material for: Assessment of quality of life in patients with surgically treated maxillofacial fractures
Source: F1000Res. 2023 Nov 6;12:483. Originally published 2023 May 11. [Version 2] doi: 10.12688/f1000research.129579.2 (PMC10988203; doi:10.12688/f1000research.129579.2)
Supplement: Peer review history

# Reviewer report: Reviewer response for version 2

**DOI:** 10.5256/f1000research.157525.r221083

**Published:** 2024-04-02

**Authors:** Gabriele Canzi

**Affiliations:** 1 Niguarda Hospital, Milan, Italy

The Authors have made an admirable calculation of the CFI score attributed to the patients; however, in the statistical analysis they chose to maintain the stratification in groups A and B based on the FISS.

 As already illustrated, the distinction between mild and moderate/severe facial trauma, using the FISS, has not been validated.

 Having consumed time to calculate the CFI score, they could use these results without also using the FISS redundantly.

 The Authors correctly divided the trauma severity groups into A and B, and the areas explored by the 20-point QOL assessment into 1 and 2. Table 3 must report "Group A and B", not "Group 1 and 2" .

Is the work clearly and accurately presented and does it cite the current literature?

Yes

If applicable, is the statistical analysis and its interpretation appropriate?

Yes

Are all the source data underlying the results available to ensure full reproducibility?

Yes

Is the study design appropriate and is the work technically sound?

Partly

Are the conclusions drawn adequately supported by the results?

Yes

Are sufficient details of methods and analysis provided to allow replication by others?

Yes

Reviewer Expertise:

Facial trauma

I confirm that I have read this submission and believe that I have an appropriate level of expertise to confirm that it is of an acceptable scientific standard.

# Reviewer report: Reviewer response for version 1

**DOI:** 10.5256/f1000research.142269.r198716

**Published:** 2023-09-04

**Authors:** Gabriele Canzi

**Affiliations:** 1 Niguarda Hospital, Milan, Italy

This paper focuses on a very interesting and extremely important topic: The personal perception on QOL of patients treated for facial trauma. Most facial traumatologists stress the radiological accuracy of their obtained results, forgetting that the real final outcome is the satisfaction and fully health of their patients.

 The work is clear, well structured, the statistics are simple but effective. I appreciated the intuition of dividing the population into Severe and Mild-to-Moderate facial injuries. This makes the results extremely convincing. Facial trauma is often not categorized in scientific research and the results leave open the question of the true homogeneity of the samples under study.

 The FISS represents a milestone, since 2006, for the severity classification of facial trauma.

 FISS stratifies the severity of facial trauma based on the anatomical sites involved, but does not distinguish the true fracture characteristics (i.e. compound or displaced fractures). Therefore it does not allow to predict the type of treatment needed (i.e. ORIF vs closed management) and the final prognosis.

  As you stated soft tissue injuries heavily influence the outcome of your study; the sot tissues consideration using the FISS (based only on the length of the wounds) is reductive.

 In the end the stratification of patients in Mild, Moderate and Severe facial trauma, using the FISS, is not statistically validated. The arbitrarily established limit value of 4.4 could be reached by a patient with LeFort II and compound nasal bone fracture (FISS=5), who can be treated with MMF and an *excellent restitutio ad integrum*.

 In 2019, the Comprehensive Facial Injury (CFI) score was proposed, which exceeded the limits of the FISS from which it derives, while maintaining its simplicity of use. Stratification of patients into Mild, Moderate and Severe facial trauma, using CFI score, was yet statistically validated and published.

 I therefore invite you to optimize your excellent work using this tool, suggesting you add a table describing the characteristics (site and type) of the fractures examined and their assigned CFI score.

Is the work clearly and accurately presented and does it cite the current literature?

Yes

If applicable, is the statistical analysis and its interpretation appropriate?

Yes

Are all the source data underlying the results available to ensure full reproducibility?

Yes

Is the study design appropriate and is the work technically sound?

Partly

Are the conclusions drawn adequately supported by the results?

Yes

Are sufficient details of methods and analysis provided to allow replication by others?

Yes

Reviewer Expertise:

Facial trauma

I confirm that I have read this submission and believe that I have an appropriate level of expertise to confirm that it is of an acceptable scientific standard, however I have significant reservations, as outlined above.

## References

[1] : The CFI score: Validation of a new comprehensive severity scoring system for facial injuries. *J Craniomaxillofac Surg* .2019;47(3) : 10.1016/j.jcms.2019.01.004 377-382 10.1016/j.jcms.2019.01.004 30709761

[2] : Understanding the relevance of comprehensive facial injury (CFI) score: Statistical analysis of overall surgical time and length of stay outcomes. *J Craniomaxillofac Surg* .2019;47(9) : 10.1016/j.jcms.2019.07.005 1456-1463 10.1016/j.jcms.2019.07.005 31375397

[3] : The Comprehensive Facial Injury (CFI) Score Is an Early Predictor of the Management for Mild, Moderate and Severe Facial Trauma. *J Clin Med* .2022;11(12) : 10.3390/jcm11123281 10.3390/jcm11123281 35743355 PMC9225200

# Reviewer report: Reviewer response for version 1

**DOI:** 10.5256/f1000research.142269.r173201

**Published:** 2023-06-08

**Authors:** Amit Sethi

**Affiliations:** 1 Department of Oral and Maxillofacial Surgery, Henry M. Goldman School of Dental Medicine, Boston University, Boston, Massachusetts, USA

The authors have done well to do “first of its kind QOL survey” for facial trauma patients. The objectives, research methodology and results have been well presented in the paper. I will highlight some of the major and minor points regarding the study.

 Major points

1. The large sample size (182 patients) with Indian patients further make the research unique and relevant.
2. The age range of patients is wide (18-70 years) and covers young as well as old patients.
3. Authors have studied patients with both mild-moderate and severe facial injury and have covered both psychosocial and esthetic-functional components.

Minor points

1. Authors have tried to establish a relation between psychological and esthetic and functional outcomes.
2. Adequate pain control which is so important in post operative course yet remains undervalued has been emphasized well by the authors
3. Poor cosmetic results and possible resultant depression and effect on QOL has also been discussed

Points that may need further clarification

1. What was the reason for doing QOL assessment after 8-12 weeks ? There is a 4 week difference in 8 and 12 weeks. 4 weeks can influence healing and QOL. How did authors account for this difference?
2. The authors do talk about post op analgesics. Were the patients prescribed post op antibiotics or in the interim 72 hrs (“most patients were treated after 72 hrs”) and did this have any bearing on outcomes?

Is the work clearly and accurately presented and does it cite the current literature?

Yes

If applicable, is the statistical analysis and its interpretation appropriate?

Yes

Are all the source data underlying the results available to ensure full reproducibility?

Yes

Is the study design appropriate and is the work technically sound?

Yes

Are the conclusions drawn adequately supported by the results?

Yes

Are sufficient details of methods and analysis provided to allow replication by others?

Yes

Reviewer Expertise:

Minor oral surgery, medicine, anesthesia, oral pathology

I confirm that I have read this submission and believe that I have an appropriate level of expertise to confirm that it is of an acceptable scientific standard.
